# Cost-Effective Large-Scale Occupancy–Abundance Monitoring of Invasive Brushtail Possums (*Trichosurus Vulpecula*) on New Zealand’s Public Conservation Land

**DOI:** 10.1371/journal.pone.0127693

**Published:** 2015-06-01

**Authors:** Andrew M. Gormley, David M. Forsyth, Elaine F. Wright, John Lyall, Mike Elliott, Mark Martini, Benno Kappers, Mike Perry, Meredith McKay

**Affiliations:** 1 Landcare Research, Lincoln, New Zealand; 2 Science and Capability Group, Department of Conservation, Christchurch, New Zealand; 3 Department of Conservation, Hokitika, New Zealand; 4 Science and Capability Group, Department of Conservation, Wellington, New Zealand; 5 Landcare Research, Palmerston North, New Zealand; University of Sydney, AUSTRALIA

## Abstract

There is interest in large-scale and unbiased monitoring of biodiversity status and trend, but there are few published examples of such monitoring being implemented. The New Zealand Department of Conservation is implementing a monitoring program that involves sampling selected biota at the vertices of an 8-km grid superimposed over the 8.6 million hectares of public conservation land that it manages. The introduced brushtail possum (*Trichosurus Vulpecula*) is a major threat to some biota and is one taxon that they wish to monitor and report on. A pilot study revealed that the traditional method of monitoring possums using leg-hold traps set for two nights, termed the Trap Catch Index, was a constraint on the cost and logistical feasibility of the monitoring program. A phased implementation of the monitoring program was therefore conducted to collect data for evaluating the trade-off between possum occupancy–abundance estimates and the costs of sampling for one night rather than two nights. Reducing trapping effort from two nights to one night along four trap-lines reduced the estimated costs of monitoring by 5.8% due to savings in labour, food and allowances; it had a negligible effect on estimated national possum occupancy but resulted in slightly higher and less precise estimates of relative possum abundance. Monitoring possums for one night rather than two nights would provide an annual saving of NZ$72,400, with 271 fewer field days required for sampling. Possums occupied 60% (95% credible interval; 53–68) of sampling locations on New Zealand’s public conservation land, with a mean relative abundance (Trap Catch Index) of 2.7% (2.0–3.5). Possum occupancy and abundance were higher in forest than in non-forest habitats. Our case study illustrates the need to evaluate relationships between sampling design, cost, and occupancy–abundance estimates when designing and implementing large-scale occupancy–abundance monitoring programs.

## Introduction

There is considerable interest in designing monitoring systems that can report on the status and trend of biodiversity at large spatial scales (i.e. internationally and globally) [1, 2]. Many countries also have national obligations to report on biodiversity change. For example, *Australia’s Biodiversity Conservation Strategy 2010–2030* [3] seeks “to stop the decline in Australia’s biodiversity”, and the New Zealand Government has required the Department of Conservation (DOC), which manages 30% of New Zealand’s land area, to report on its achievements in reducing the rate of biodiversity loss [4]. Reporting on biodiversity change requires biodiversity monitoring (defined as the systematic acquisition of information) [5] and reporting systems that provide unbiased assessments of biodiversity change [6–9]. Current international efforts to design biodiversity monitoring programs are focusing on the ‘indicator’ variables that might be measured [2, 10–12], but remarkably little consideration has been given to the costs of large-scale biodiversity monitoring [11, 13]. A major impediment to the implementation of large-scale biodiversity monitoring and reporting programs is their perceived high cost [14, 15]. The total cost of a biodiversity monitoring system will largely be determined by the number of locations that are sampled, the labour and field allowance costs for each location (‘variable costs’), transport and equipment costs per location (‘fixed costs’), plus the overarching staff and associated infrastructure costs required to manage the data collation, storage and reporting activities (‘overheads’) [16–18]. Of particular importance will be how the fixed and variable costs change with increasing sampling intensity (i.e. number of sampling locations and spatial coverage).

Two key indicators of status and trend in a taxon are occupancy (proportion of sites used) and abundance (number or density per site) [19, 20], and both have been suggested as Essential Biodiversity Variables that could be monitored worldwide [12]. Occupancy can be defined as the proportion of sampling locations used by a taxon and is estimated from presence/absence data (or more correctly, detection/non-detection data) collected at sampling locations [20, 21]. Occupancy models can also be used to estimate the spatial distribution of a species [22, 23]. Abundance is the number of individuals present within an area of interest, but is difficult to estimate accurately and precisely for animals [19]. Hence, indices of relative abundance (‘any measurable correlative of density’) [24] are more commonly used as the basis for decision-making in the management of animal populations [25]. Although the quantities occupancy and abundance are interrelated [26, 27], many studies report only one quantity. A substantially richer picture of the status and trend of taxa of interest can be obtained by jointly reporting occupancy and abundance [26–28].

Invasive species can have substantial ecological, social and economic impacts [29–32], such as causing biodiversity decline (including extinctions) [33] and ecosystem change [34]. Hence, large-scale biodiversity monitoring schemes will often include invasive species. Increases in occupancy and abundance of invasive species are usually considered undesirable [30], with considerable effort often expended attempting to reduce the occupancy and abundance of invasive species [35].

Here, we describe the development of a cost-effective monitoring system for reporting on changes in occupancy and abundance of an ecologically and economically destructive invasive species, the brushtail possum (*Trichosurus vulpecula*), on New Zealand’s public conservation land. Specifically, we use data collected in the phased implementation of the large-scale monitoring program to evaluate the consequences of altering sampling effort for possum occupancy–abundance estimates and the annual cost of the monitoring program. We discuss how our approach can assist other practitioners and researchers in the design and implementation of cost-effective large-scale occupancy and abundance monitoring and reporting programs.

### Invasive brushtail possums in New Zealand

**Fig 1:**
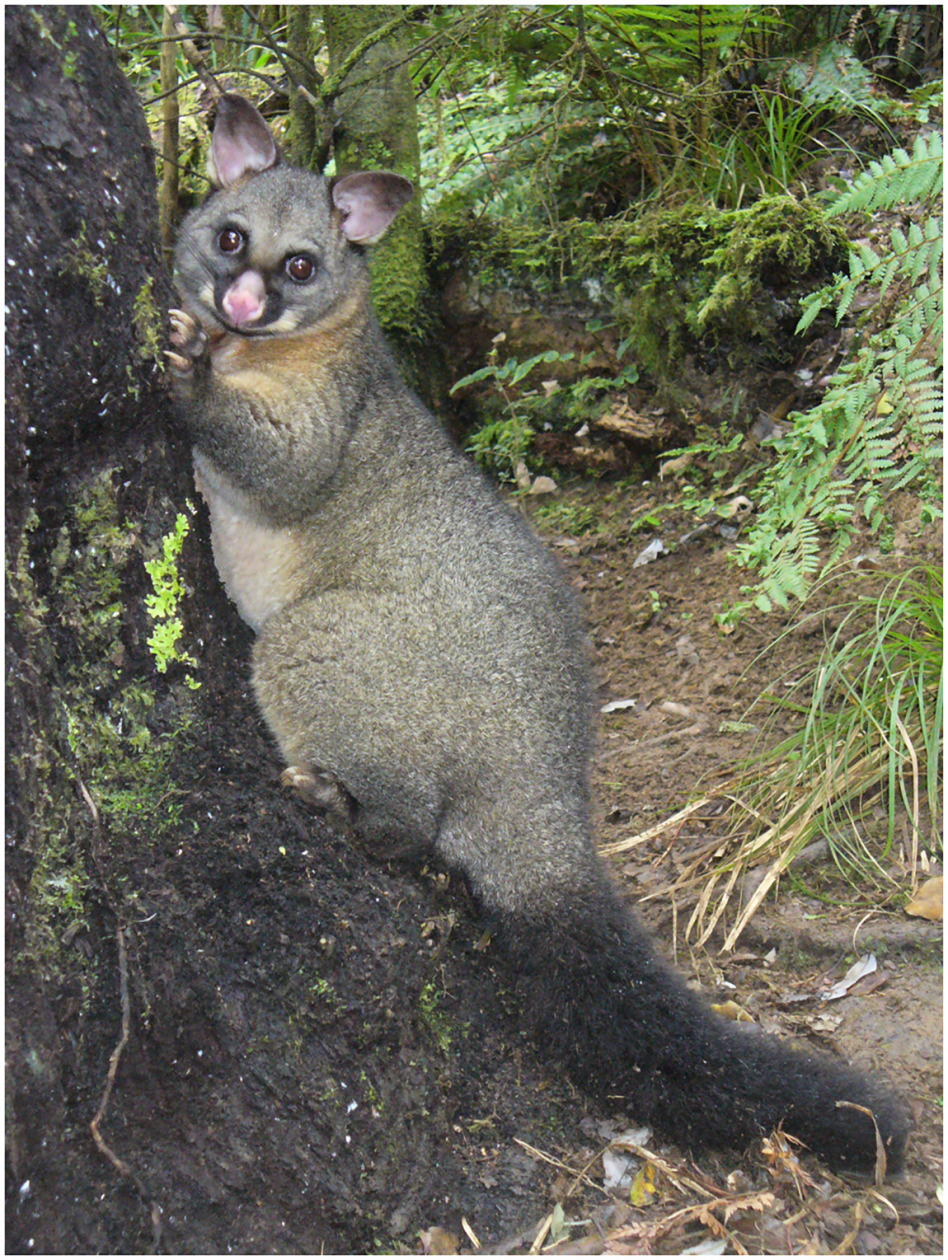
The brushtail possum was introduced from Australia to New Zealand and is now a major pest. (Photo: Grant A. Morriss.)

New Zealand has been considered an exemplar of island vulnerability to invasion by alien biota [36, 37]. At European colonisation, New Zealand had no native land mammals other than two species of bat [38]. Settlers deliberately or inadvertently introduced numerous species of mammal into New Zealand, many of which have established self-sustaining populations [38]. The brushtail possum (Fig 1) is a nocturnal and omnivorous Australian marsupial (adult body mass = ca. 3 kg) that was deliberately introduced into New Zealand to establish a fur trade [39, 40]. The possum is thought to be widespread in the North, South and Stewart islands of New Zealand, and is considered New Zealand’s most important vertebrate pest of conservation and agriculture [40]. Possums can defoliate and kill tree species in some circumstances [41, 42], and prey on the eggs and nestlings of endangered birds [43]. Possums are also maintenance hosts of bovine tuberculosis, which poses a threat to the health of domestic livestock in New Zealand [44]. Considerable effort has been expended protecting conservation and agricultural values by controlling possums with toxins [45, 46] and leghold traps [47].

## Materials and Methods

### Department of Conservation’s biodiversity monitoring and reporting system

DOC is the central government organisation charged with conserving the natural heritage of New Zealand. This mandate requires DOC to know where natural heritage outcomes are being achieved and how management interventions can be used to improve poor outcomes. The desired outcome of conserving natural heritage has been defined as maintaining ecological integrity [48]. Reporting on progress towards achieving this outcome (*sensu* McDonald-Madden et al. [8]) requires an inventory and monitoring program to provide unbiased, repeatable ecological-integrity indicator estimates for all public conservation land (i.e. the 8.5 million ha of land managed by DOC).

**Fig 2:**
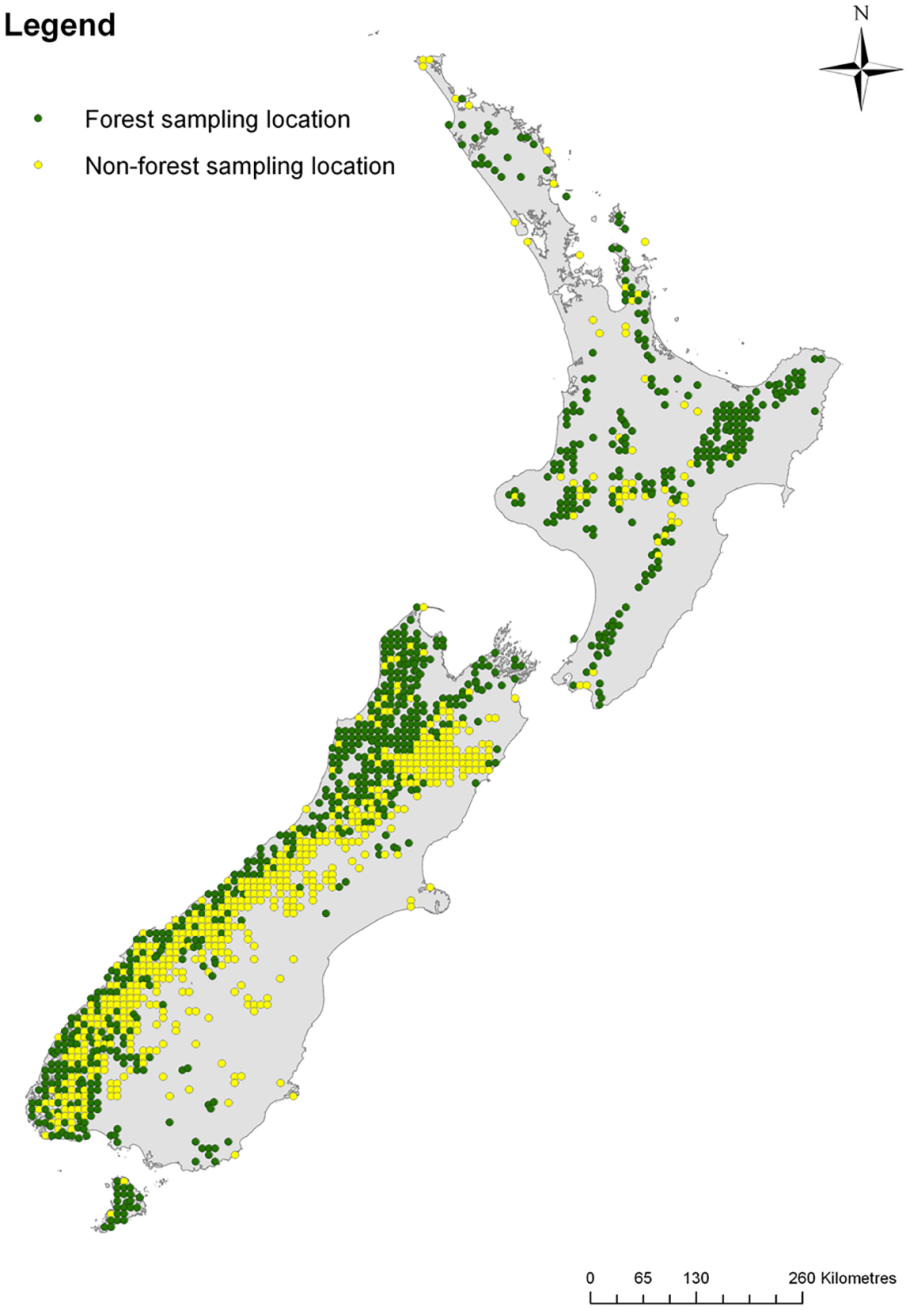
Sampling locations in the Department of Conservation’s Biodiversity Monitoring and Reporting System. The 1354 sampling locations are located at the vertices of an 8-km grid superimposed over New Zealand’s public conservation lands.

The Biodiversity Monitoring and Reporting System (BMRS) was developed in order to report on three natural heritage priority indicators: Indigenous dominance, Species occupancy, and Ecosystem representation [18]. Building on the network of carbon monitoring plots established in New Zealand during the early 2000s [49], one component of the BMRS involves monitoring at sampling locations located at the vertices of an 8-km grid superimposed over New Zealand’s public conservation land, including North, South and Stewart islands and offshore islands (i.e. a spatially unbiased monitoring system; Fig 2). The monitoring gathers information on five measures: Size-class structure of canopy dominants; Representation of plant functional types; Distribution and abundance of exotic weeds; Distribution and abundance of exotic pests; and Assemblages of widespread animal species—Birds. There are currently 1354 sampling locations on public conservation land (786 in forest habitat and the remainder in non-forest habitat): when fully implemented, approximately 271 randomly selected sampling locations would be monitored annually on a rolling five-year cycle. For full details of the sampling methods, see Allen et al. [18].

**Fig 3:**
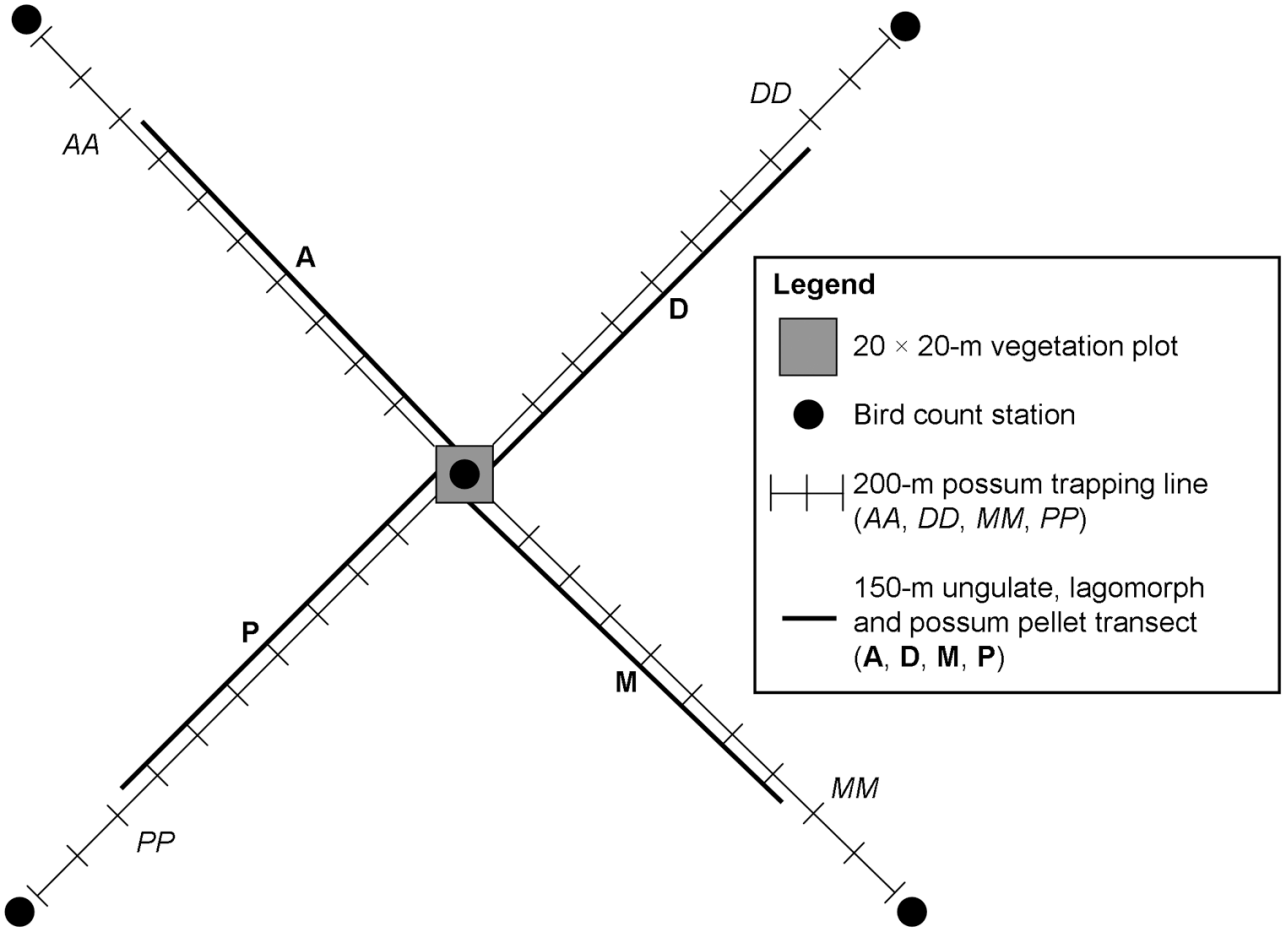
Spatial design of invasive mammal monitoring conducted at each sampling location [18]. Brushtail possum trapping occurred along each of the four 200-m trap-lines (AA, DD, MM and PP), and the presence/absence of possum faecal pellets was recorded in each of the 30 quadrats located along the four 150-m ungulate/rabbit/hare pellet transects (A, D, M and P). Birds were monitored at each of the bird monitoring stations, and vegetation was monitored at the central 20 × 20-m vegetation plot.

Each sampling location (Fig 3) is permanently marked to enable repeat sampling [18]. Vegetation measurements are all made within a fixed 20 × 20-m plot (0.04 ha). Data on invasive mammals (i.e. brushtail possums, a variety of ungulate taxa and two species of lagomorphs) and common birds are collected within a much larger area (331 × 331 m; 10.96 ha), using a design that radiates from the edges of the central vegetation plot (Fig 2). Standardised field sampling protocols were used for the vegetation, mammal and bird surveys [49–51].

### Pilot study and phased implementation of the BMRS

There are three resource requirements for any monitoring program [52]: funding, time, and the availability of adequate technical expertise. To test the feasibility and cost of implementing the proposed BMRS [18, 51], a pilot study was conducted at 18 sampling locations in the 2008–09 austral summer. The pilot study revealed that the two nights required for possum monitoring (see below) was a major determinant of costs, time investment, and the availability of adequate technical expertise [18]. Reducing possum monitoring from two nights to one night would reduce the cost of the BMRS because staff would stay two days rather than three days at each sampling location. Monitoring for two days rather than three days would mean that fewer field teams would be needed to complete the fixed number of sampling locations within a field season, resulting in a reduced labour cost through fewer days spent in the field. Furthermore, fewer people would need to be trained to meet the minimum standard required to conduct the possum field monitoring. However, there were insufficient data to make an informed decision on the effects of monitoring for one night rather than two nights on estimates of brushtail possum occupancy and abundance. Based partly on the results of the pilot study, it was decided that the implementation of the BMRS would continue using those methods tested in the pilot study, but that a benefit–cost analysis of monitoring possums for one night rather than two nights would be conducted at the completion of the first two years of implementation. The phased implementation of the BMRS was designed so that 80 randomly selected forest locations and 80 randomly selected non-forest locations would be sampled in the 2011–12 and 2012–13 field seasons (i.e. October–March; Fig 2), before increasing to 271 locations per field season from the 2013–14 field season.

### Field methods for monitoring brushtail possums

**Fig 4:**
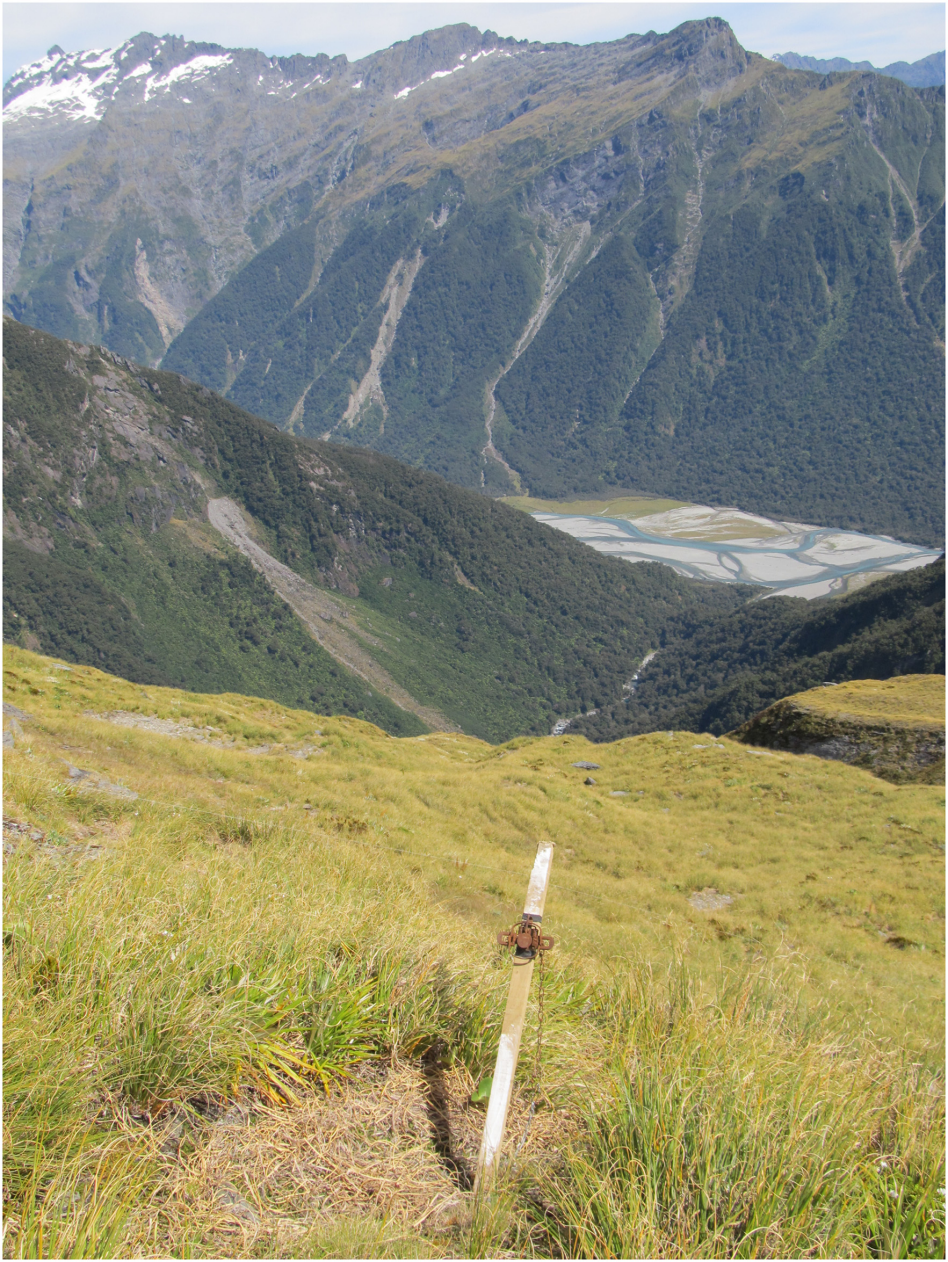
Trap set on a brushtail possum trap-line at a sampling location in non-forest habitat. (Photo: David M. Forsyth.)

Two types of data used to estimate possum occupancy and abundance were collected at each of the sampling locations. First, the presence/absence of possum faecal pellets was recorded in each of 30 circular plots of 1-m radius located at 5-m intervals along four faecal pellet transects (A, D, P and M in Fig 3) radiating from the four corners of the 20 × 20-m vegetation plot that was the focus of each sampling location. Ungulate and lagomorph faecal pellets were counted along these transects and used to estimate national occupancy and relative abundances for these taxa [50, 53], and the time taken to collect these pellet data was not identified as a constraint on field sampling. Hence, we did not fully explore the consequences of altering the faecal pellet sampling effort. Second, 10 No. 1 double-coil-spring leghold traps were set (Fig 4) at 20-m intervals along each of four trap-lines radiating from the corners of the vegetation plot and parallel to the pellet lines (AA, DD, MM and PP in Fig 3). Traps were set for two consecutive nights without rainfall (to standardise trapping efficiency), following a national protocol [54]. Traps were checked within 12 hours of sunrise and any captured possums humanely euthanased. For further details see [50] and [54].

One team of two people conducted the bird and mammal pest (including possum) monitoring during the one visit to each sampling location during the field seasons (October–March) of 2011–12 and 2012–13. (Another team of two people conducted the vegetation monitoring, sometimes concurrently with the bird and mammal pest monitoring.) Based on information collected during the 2011–12 and 2012–13 field seasons, it was determined that a field team of two people would usually be able to complete the bird and mammal pest monitoring in two days if each member of the team worked for 10 hours on both days rather than 8 hours on each of three days. Reducing monitoring from three to two days would also enable better coordination between the vegetation and fauna monitoring teams (i.e. both teams could travel together more often).

### Costs of monitoring possums for one night and two nights

Since the number of locations to be sampled annually has already been determined (i.e. 271 annually on a five-year rotation) [18], the costs of equipment and travel to and from each plot would not change if trapping effort was reduced from two nights to one night (i.e. these are ‘fixed costs’). The rugged and remote nature of the sampling universe meant that the majority of sampling locations required a helicopter to be chartered to drop off and pick up the field team and their equipment, and the cost of these flights was collated from invoices. Vehicle transport costs were estimated by multiplying the kilometres driven by the field teams by the DOC’s per-kilometre rate. Only the labour-related costs would change if trapping was to be reduced from two nights to one night (i.e. ‘variable costs’). We used an hourly labour rate of $22.50 (all costs are in 2013 NZD), a daily field allowance of $21.00 and a daily food cost of $22.61 in our analyses.

We also accounted for the effect of bad weather on the time to complete sampling. Trapping for one night rather than two nights means that only a one-night (rather than two-night) window of fine weather is needed, and hence over a field season (i.e. October–March) there will be more opportunities to conduct sampling and fewer teams may be needed. Field teams work weekends if the weather is suitable, but do not work during a 10-day period that includes Christmas Day and New Year’s Day. There are potentially 172 field days available for conducting sampling during a field season but, based on monitoring of systematically located vegetation plots for carbon accounting, approximately 30% of these days are likely to be unsuitable for possum monitoring due to bad weather [18].

### Statistical analyses

In all analyses, ‘sampling location’ was the unit of replication [55], with the four trap-lines and four pellet-lines treated as repeat samples within each sampling location (i.e. surveys of spatial subunits). We define occupancy as the proportion of sampling locations used by possums (*sensu* ref [56]). We did not attempt to estimate the area of public conservation land used by possums, because this would require the area sampled by the four trap-lines and four pellet-transects to be estimated (see Discussion).

Information on occupancy was obtained by summarising trap data by trap-line, such that the observed presence or absence of trapped possums on trap-line *j* at sampling location *i* was indicated by *X*_*i*, *j*_ = 1 or 0, respectively, for *j* = 1–4 (i.e. corresponding to the four trap-lines AA, DD, MM and PP). Similarly, the possum faecal pellet data were summarised for each transect such that the presence or absence of pellets at sampling location *i* and pellet transect *j* was indicated by *X*_*i*, *j*_ = 1 or 0, respectively, for *j* = 5–8 (corresponding to the four pellet transects A, D, P and M). The relative abundance of brushtail possums along each of the four trap lines was estimated following the Trap Catch Index (TCI) protocol [54]. Briefly, the total number of possums caught is divided by the number of trap nights minus 0.5 trap nights for each non-target capture and sprung but empty trap [57], and that number is multiplied by 100% [54]. The TCI is an index of relative abundance that has been demonstrated to be positively related to ‘true’ possum abundance [58, 59]. *Y*_*i*, *j*_ denotes the observed TCI at sampling location *i* on trap-line *j*, for *j* = 1–4 (corresponding to the four trap-lines AA, DD, MM and PP). Information from the four pellet transects was not used to inform possum abundance.

The data were analysed using a joint occupancy–abundance model to account for overdispersion and imperfect detection [28, 60]. The true presence of possums in the sampling location was modelled as a random variate from a Bernoulli distribution with probability *ψ*, the probability of occupancy:

$$z_{i}\sim \text{Bern}(\psi )$$ (1)

For each survey method (i.e. faecal pellets and traps), there is a probability of detection per line conditional on the site being occupied. The observed presences/absences were modelled as:

$$X_{i}{}_{,j}\sim \text{Bern}(z_{i}\times p_{j})$$ (2)

where *p*_*j*_ is the detection probability for each survey (recall *j* = 1–4 denotes trapping lines and *j* = 5–8 denotes faecal pellet transects). The model allowed detection probabilities to differ between the trapping and pellet methods, but they were constant within each method.

TCI values for each trap-line were modelled using a zero-inflated model to account for excess zeros due to true absences [28] as:

$$Y_{i}{}_{,j}\sim \text{Poisson}(z\lambda_{i})$$ (3)

where *z*_*i*_ is the estimated presence/absence of possums at sampling location *i* as estimated earlier by the occupancy component of the model, and *λ* is the mean TCI at a sampling location, conditional on it being occupied by possums. The mean TCI across all sampling locations (i.e. occupied and unoccupied) is given by *zλ* = *λ* × *ψ*.

We first explored the consequences of reducing trapping effort from two nights to one night on estimates of occupancy (*ψ*) and relative abundance (*zλ*) using data from two nights versus the first night of trapping only. We estimated occupancy (*ψ*) and relative abundance (*zλ*) for all the public conservation land and then separately for forest and non-forest habitat (i.e. *λ* and *ψ* could differ for forest and non-forest sampling locations), with sampling locations defined as forest or non-forest habitat using the Land Cover Database [61] supplemented with descriptions made by the field staff conducting monitoring.

We next explored the consequences of reducing the number of trap-lines used at each sampling location for estimates of relative abundance and occupancy, again using data from two nights versus the first night of trapping. For each specified number of trap-lines, trap-line data were sampled without replacement from each sampling location, and parameters were estimated as previously. This was done 100 times to obtain a range of estimates of occupancy and mean TCI.

Our occupancy–abundance models were fitted in a Bayesian framework using OpenBUGS 3.3.1 [62]. Bayesian inference was based on posterior distributions of parameters; these are the conditional distributions of parameters, given data. Bayesian inference requires the specification of prior distributions of parameters; if the prior distribution is diffuse and/or if the sample size is large, then the posterior distribution is primarily a reflection of the likelihood function, so that Bayesian inference and classical Frequentist inference will produce similar results [63]. We used diffuse prior distributions of Beta(1,1) for occupancy *ψ* and detection probabilities *p*_*j*_ (i.e. there was equal probability of *ψ* and *p*_*j*_ being anywhere between 0 and 1). A similarly uninformative prior distribution of Normal (0,100) was used for the log of *λ*. Although we could have used more informative priors, we chose to let the observed data determine the posterior distributions for occupancy and detection. Two Markov chains were constructed using different initial values and run for 10,000 burn-in iterations to ensure convergence. These burn-in samples were discarded and the algorithm run for a further 50,000 iterations before the two chains were combined to provide a sample of 100,000 values from the joint posterior distribution of each parameter. All parameter estimates are presented with 95% credible intervals (CIs), and the relative precision of the estimates was compared using the coefficient of variation (CV; standard deviation divided by the mean of the posterior distribution).

Finally, we conducted power analyses to determine the number of sampling locations that would provide 80% power of detecting a 10% change in TCI between time 1 (*t*_1_) and time 2 (*t*_2_). We also determined the percentage change in TCI that could be detected for specified levels of power, assuming sampling in all 786 forest sampling locations at *t*_1_ and *t*_2_, and also for half (i.e. 393) of the forest sampling locations at *t*_1_ and *t*_2_. We used the coefficient of variation (CV) (estimated using the one-night and two-night trapping data) in the power analyses. The CV is the standard deviation of the TCI divided by the mean of the TCI. Our power analyses were performed using the base function *power*.*t*.*test* in R [64].

### Data availability

Data are available from the Dryad Digital Repository: http://datadryad.org/review?doi=doi:10.5061/dryad.fn6h0.

### Ethics statement

Under New Zealand law, Institutional Animal Ethics Committee approval was not required for this study because possums were trapped for management purposes, not for research, testing or teaching. Permission to trap possums at the 164 sampling locations was provided by the New Zealand Department of Conservation, which manages New Zealand’s public conservation land. All possums trapped in this study were handled in accordance with the national possum monitoring protocol [54]. Traps were checked within 12 hours of sunrise, as required by the Animal Welfare Act 1999. Trapped possums were killed immediately and humanely with a sharp blow to the cranium [50, 54].

## Results

Possum occupancy and relative abundance was assessed at 164 sampling locations in 2011–12 and 2012–13: 85 sampling locations in forest habitat and 79 sampling locations in non-forest habitat (S1 Fig). Only 16% of sampling locations did not have all four trap-lines and four pellet lines surveyed, mostly due to the presence of dangerous terrain.

### Effects of trapping for one and two nights on variable costs

Approximately 271 sampling locations will be measured annually when the monitoring is fully implemented. Two nights of trapping requires 813 field days per annum (i.e. 271 × 3 days), whereas one night of trapping requires 542 field days (i.e. 271 × 2 days). After accounting for bad weather, 120 days are available for each team each year, and therefore seven two-person teams would be required to measure 271 sampling locations per field season if trapping was conducted for two nights, whereas five two-person teams would be required if trapping was reduced to one night. Reducing trapping effort from two nights to one night along four trap-lines reduces the costs of monitoring by $267.22 (5.8%) per sampling location (Table 1).

**Table 1:** Change in the cost of monitoring if brushtail possum trapping is reduced from two nights to one night.

Item		Cost per unit	Possum sampling effort			
			Two nights’ trapping		One night’s trapping	
			Units	Cost	Units	Cost
**Fixed costs**	**Transport**	$2,943.00 per location^a^	1	$2,943.00	1	$2,943.00
	**Equipment**	$335.00 per location	1	$335.00	1	$335.00
**Variable costs**	**Labour**	$22.50 per hour	2 × 8 h × 3 d = 48 h	$1,080.00	2 × 10 h × 2 d = 40 h	$900.00
	**Field allowance**	$21.00 per person per day	2 × 3 d = 6 d	$126.00	2× 2 d = 4 d	$84.00
	**Food**	$22.61 per person per day	2 × 3 d = 6 d	$135.66	2× 2 d = 4 d	$90.44
**Total costs**				$4,619.66		$4,352.44

Units: ‘location’ = sampling location, ‘d’ = day and ‘h’ = hour. All costs are in 2013 NZD.

^a^ This amount consists of $498.00 and $2,445.00 for vehicle and helicopter costs, respectively.

The costs shown are those incurred during the 2011–12 and 2012–13 field seasons.

Weather plays a critical role in determining the order in which locations are sampled within a field season, but adjacent locations are, wherever possible, sampled sequentially in order to minimise transport costs.

### Effects of trapping for one and two nights on occupancy–abundance estimates

**Fig 5:**
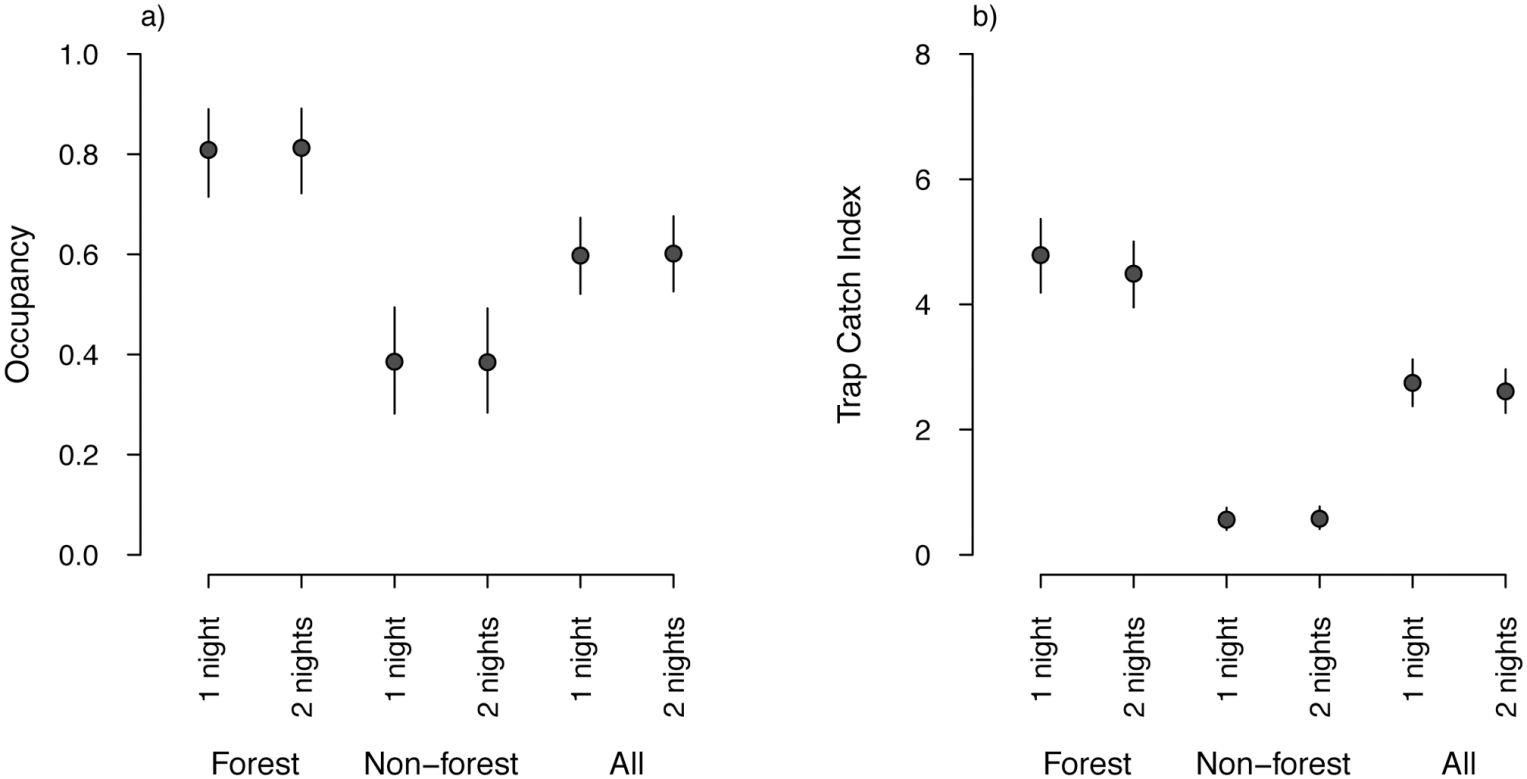
Effects of reducing sampling effort on estimated brushtail possum occupancy and relative abundance. (a) Occupancy. (b) Relative abundance (Trap Catch Index). There were 85 sampling locations in forest habitat and 79 sampling locations in non-forest habitat. Vertical bars indicate 95% credible intervals.

Estimates of mean possum occupancy were invariant to the number of trap nights with respect to bias and precision for both forest and non-forest sampling locations, and when pooled across all sampling locations (Fig 5). When trapping was reduced from two nights to one night, the CV of the mean occupancy increased only slightly: from 5.4% to 5.6% in forest habitat and from 13.8% to 14.0% in non-forest habitat. Estimates of mean TCI were relatively higher (and were estimated with less precision) when trapping was conducted for one night compared with two nights. This effect was more pronounced in forest habitat, where mean TCI values increased from 4.5% (CV = 6.0) to 4.8% (CV = 6.3) when the number of nights of trapping was reduced from two nights to one night (Fig 5).

The small increase in the variability when reducing trapping from two nights to one night means that the number of locations required for detecting a specified change in possum abundance increases. To have 80% power of detecting the desired relative change in mean TCI of 10% (i.e. from a TCI of 5.0 to 5.5) requires repeat measures at 225 sampling locations if trapping is conducted for two nights and at 250 sampling locations if trapping is conducted for one night. From the full 786 sampling locations in forest habitat, the minimum level of change in abundance able to be detected with a specified level of power increased slightly. For example, there is an 80% chance of detecting a relative change in the mean TCI of 5.4% from two nights of trapping compared with a 5.7% change from one night of trapping (Table 2). The relative change in TCI that would be detected with 95% power by repeat sampling of the 786 sampling locations is 6.9% and 7.3% with two nights and one night of trapping, respectively.

**Table 2:** Change in the relative abundance of possums (i.e. Trap Catch Index) able to be detected for three levels of power for forest locations for two nights and one night of trapping.

Power (%)	Two nights’ trapping		One night’s trapping	
	Absolute change	% change	Absolute change	% change
**80**	0.27	5.4	0.29	5.7
**90**	0.31	6.2	0.33	6.5
**95**	0.34	6.9	0.36	7.3

Values are for repeat measurements at *n* = 786 forest sampling locations and a Trap Catch Index of 5 at time 1.

### Effects of reducing trap-lines rather than sampling locations on occupancy–abundance estimates

**Fig 6:**
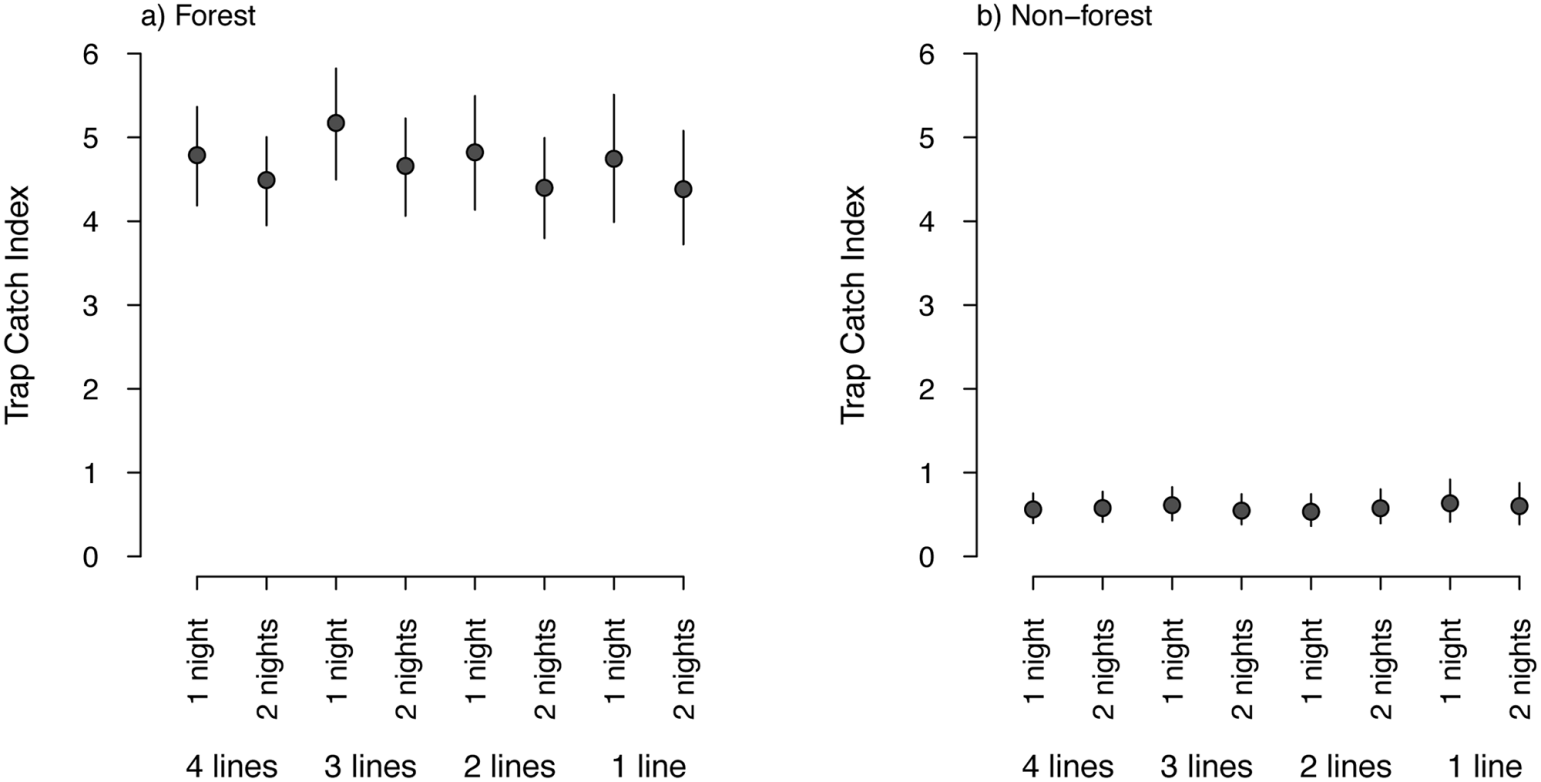
Effects of reducing sampling effort on estimated brushtail possum relative abundance (Trap Catch Index). (a) Forest habitat (*n* = 85 sampling locations). (b) Non-forest habitat (*n* = 79 sampling locations). Abundances are shown for varying numbers of trap-lines (one to four per sampling location) and trap nights (one or two). Vertical bars indicate 95% credible intervals.

Reducing the number of trap-lines used at each sampling location had little effect on estimated occupancy, but had a stronger effect on estimated abundance. For forest locations, mean estimates of possum abundance from one night of trapping were consistently higher than estimates from two nights of trapping for any fixed number of trap-lines (Fig 6). The current sampling effort of four trap-lines per sampling location resulted in a CV of the mean TCI of 6.0% in forest locations, whereas the minimum sampling effort (i.e. one trap-line set for one night) increased the CV of the mean TCI to 8.2%. Despite this increase, the CV of the mean TCI is relatively invariant to changing effort (i.e. reducing lines and or nights), because it is primarily affected by the total number of sampling locations (held constant in this analysis).

**Fig 7:**
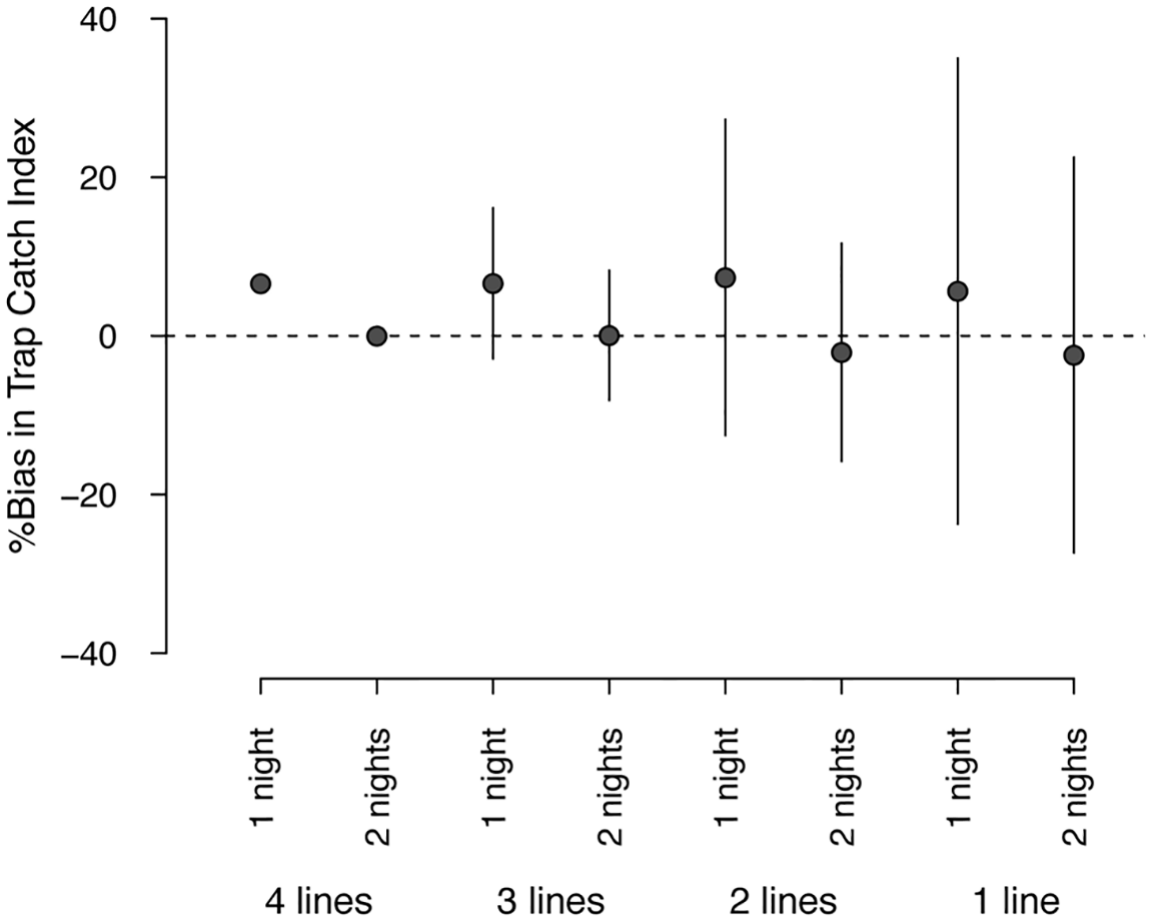
Effects of reducing sampling effort on the bias of estimated brushtail possum relative abundances (Trap Catch Index). Biases are shown for varying numbers of trap-lines (one to four per sampling location) and trap nights (one or two) relative to the current sampling effort (four trap-lines set for two nights). Circles and vertical lines indicate the mean and potential range of relative bias in estimates of Trap Catch Index as a result of subsampling from four trap-lines per sampling location.

More importantly, reducing the number of trap-lines increases the potential for obtaining estimates that are biased relative to the current sampling effort (Fig 7). In forest locations, when one night of trapping was used, the mean bias was only ca. 6% compared with two nights of trapping, but the range of potential bias increased greatly when fewer trap-lines were used per sampling location. At the minimum sampling effort (one trap-line set for one night), the relative bias of TCI estimates ranged from −24% to 35% compared with the TCI from the current sampling effort estimates of possum abundance in forest habitat (Fig 7), corresponding to estimated mean TCIs of between 3.3% and 6.0%.

## Discussion

### Cost-effective monitoring of invasive brushtail possums on New Zealand’s public conservation land

Reducing trapping effort from two nights to one night along four trap-lines reduced the estimated costs of monitoring by 5.8% per sampling location due to the reduced costs of labour, food and allowances. If 271 sampling locations were monitored annually, then trapping for one night rather than two nights would provide an annual saving of about $72,400. Trapping possums for one night rather than two nights also means that approximately 542 rather than 813 field days would be needed for sampling. Another important, but difficult to quantify, efficiency gain resulting from reducing trapping from two nights to one night is that there would be many more suitable ‘weather windows’ (of one fine night versus two fine nights) during which possum monitoring could be undertaken.

Reducing trapping from two nights to one night had a negligible effect on estimated national possum occupancy in terms of both variance and bias, but resulted in higher and less precise estimates of relative abundance, especially in forest locations. The ability to detect differences in occupancy was not greatly affected by a reduction from two nights to one night of trapping, although the small increase in variance for relative abundance means a small reduction in the ability to detect a given level of change in possum occupancy and abundance. Importantly, reducing from two nights to one night of trapping provides more than 80% power to detect the desired relative change of 10% in possum abundance over a five-year period (i.e. the time between remeasurements). Indeed, repeat sampling of the 786 forest locations using only one night of trapping has 95% power to detect a relative change in possum abundance of 7.3%.

The similar estimates of occupancy for one and two nights of trapping are partly due to the additional pellet information used to estimate occupancy. The possum pellet information is recorded when ungulate pellets are counted, and there is no significant additional cost associated with this activity. Estimated occupancy decreased from 0.60 (when estimated using data from four trap-lines and four pellet-lines) to 0.43 (when estimated using data from only the four trap-lines), highlighting the importance of using both detection methods for the estimation of possum occupancy.

Reducing the number of trap-lines did not change the estimates of possum occupancy in terms of bias or precision, due primarily to the high value of the information from the faecal pellet plots. Despite the reduction in trap-lines only having a small effect on the precision of estimated possum abundances, large biases of unknown direction (i.e. positive or negative) could occur (Fig 7). The potential bias increased with a reduction in the number of trap-lines, and could be as high as 35% when only one trap-line was set. Any reduction in the number of trap-lines would result in estimates of TCI that have a similar level of precision, yet are likely to be highly biased, potentially resulting in changes or differences in relative abundance being reported that are merely an artefact of sampling.

Based on the analyses reported here, possum trapping effort was reduced from two nights to one night for the monitoring of 271 sampling locations during the 2013–14 field season, and will continue at that level in future years. However, once all 1354 sampling locations on public conservation land have been sampled once, it would be prudent to evaluate how reducing the number of sampling locations remeasured annually affects estimates of possum occupancy–abundance estimates and the ability to detect the desired changes in these measures. Based on the current costs of monitoring a sampling location (Table 1), reducing the number of sampling locations measured annually would substantially reduce the cost of the program. Our results also suggest that the national possum monitoring protocol, which recommends that the TCI is estimated from either two or three nights of trapping [54], could be revised to one night. However, estimates of possum abundance made using the TCI are used for a variety of management and reporting purposes in New Zealand, and we recommend that the sampling effort (i.e. number of trap-lines and number of nights of sampling) be optimised for each purpose [65].

### Brushtail possum occupancy and abundance on New Zealand’s public conservation land

The purpose of the monitoring program reported here is to provide unbiased estimates of brushtail possum occupancy and relative abundance on New Zealand’s public conservation land. Estimates of occupancy vary with many factors, including the size of the sampling unit [66], the home-range size of the taxon being monitored [66], and the detection probabilities of the taxon given the monitoring methods ([56], this study). We estimated the proportion of sampling locations used by possums, rather than the area of public conservation land used by possums: the latter would require the area sampled by the four trap-lines and four pellet-transects to be estimated [66]. The area sampled by leg-hold traps has been estimated in forest habitat [59] but not in non-forest habitat; the area sampled by pellet counts has not been estimated in any habitat. We assumed that the presence of one or more possum pellets at a sampling location indicated that one or more possums were present to be captured (detected) in leg-hold traps. However, possum pellets could take many months to decay, and hence this assumption may sometimes have been violated. We emphasise that our estimates of possum occupancy, which are unbiased due to the systematic selection of sampling locations on an 8×8-km grid, are therefore conditional on the monitoring design used within sampling locations.

Our analyses confirm that possums commonly occupy forested habitat on public conservation land [40]. However, relatively little is known about possums in New Zealand’s non-forested habitat, and our results indicate that possums are much less commonly present and at lower abundances in non-forested than forested habitats on public conservation land. Even within forests, possum abundances were considerably lower than expected based on previous studies (see review by Efford [67]). The lower-than-expected abundances of possums in forested and non-forested habitats on public conservation land may be due to several causes, such as previous studies estimating possum abundances at locations known to contain possums or uncontrolled populations rather than at an unbiased sample of locations. For example, Glen et al. [68] used the TCI to estimate brushtail possum abundances in a non-forest habitat “where populations were undisturbed and so likely to be at carrying capacity”. The unbiased possum occupancy and relative abundances reported here for New Zealand’s public conservation land provide important baseline information for comparing against future assessments and for evaluating the effects of possum management activities [53, 69].

### Designing cost-effective large-scale occupancy–abundance monitoring programs

The need for monitoring and research programs to evaluate their cost-effectiveness to ensure that limited resources are sensibly allocated has been well articulated [14, 16, 52, 70]. However, to our knowledge there are no published examples of this being done for a large-scale occupancy–abundance monitoring program, such as is being implemented for selected taxa by the New Zealand DOC [18]. Our approach to initial uncertainty about the relationship between the sampling design (and hence costs) and the accuracy and precision of occupancy–abundance estimates generated by a small-scale pilot study [18] was to conduct a phased implementation of the monitoring program that collected the information required to robustly evaluate those relationships. This approach could be considered ‘adaptive monitoring’ (*sensu* Lindenmayer and Likens [71]).

Our study has several other lessons for the design of occupancy–abundance monitoring programs at any spatial scale. First, increasing sampling effort eventually leads to negligible increases in the accuracy and precision of occupancy–abundance estimates. For brushtail possums in New Zealand, varying either the number of trap-lines sampled at each sampling location and/or the number of nights sampled changed the precision of occupancy and abundance estimates little relative to altering the number of sampling locations. This result occurred because sampling location was the unit of replication in these analyses [27, 28, 60]. Second, the costs of increasing field sampling do not increase monotonically but rather via a series of ‘steps’. The key costs of sampling brushtail possums in New Zealand’s BMRS are (i) moving between sampling locations (because helicopters are commonly used), and (ii) staying an additional night at a sampling location (due to increased labour costs). Understanding these steps in cost is critical to forecasting and optimising the costs of large-scale monitoring programs. Third, there are logistical and capacity issues that cannot easily be framed as a financial cost of sampling. The phased implementation identified that the weather window required to complete two nights of possum monitoring following the national protocol occurred much less frequently than if trapping was conducted for only one night. This constraint was not identified in the small-scale pilot study [18]. A pilot study and/or phased implementation will enable such issues and their associated costs to be identified.

## Conclusion

Our study serves as a practical guide for others considering implementing large-scale biodiversity monitoring programs, whether for one taxon or many taxa. Conducting a pilot study enabled identification of significant labour and logistical savings arising from reducing monitoring of possums from two-night sampling to one-night sampling. The phased implementation of the monitoring program allowed the key data to be collected (and benefit–cost analyses to be performed) before the program was fully implemented. More generally, our study illustrates how additional sampling may have little additional effect on estimates of occupancy and abundance for a taxon. The challenge for large-scale monitoring programs is to identify these situations so that sampling can be optimised and the costs of the programs reduced. Our study highlights the need to evaluate relationships between sampling design, cost, and occupancy–abundance estimates when designing and implementing large-scale monitoring programs.

## Supporting Information

S1 Fig The 164 sampling locations (*n*_forest_ = 85, *n*_non-forest_ = 79) at which brushtail possum monitoring was conducted during the phased implementation of the 2011–12 and 2012–13 field seasons. (PDF)
