# Supplementary material for: Cost-Effective Large-Scale Occupancy–Abundance Monitoring of Invasive Brushtail Possums (Trichosurus Vulpecula) on New Zealand’s Public Conservation Land
Source: PLoS One. 2015 Jun 1;10(6):e0127693. doi: 10.1371/journal.pone.0127693 (PMC4452217; doi:10.1371/journal.pone.0127693)

Supporting Information

Figure S1. The 164 sampling locations (n_{forest} = 85, $n_{\text{non-forest}}$ = 79) at which brushtail possum monitoring was conducted during the phased implementation of the 2011–12 and 2012–13 field seasons.

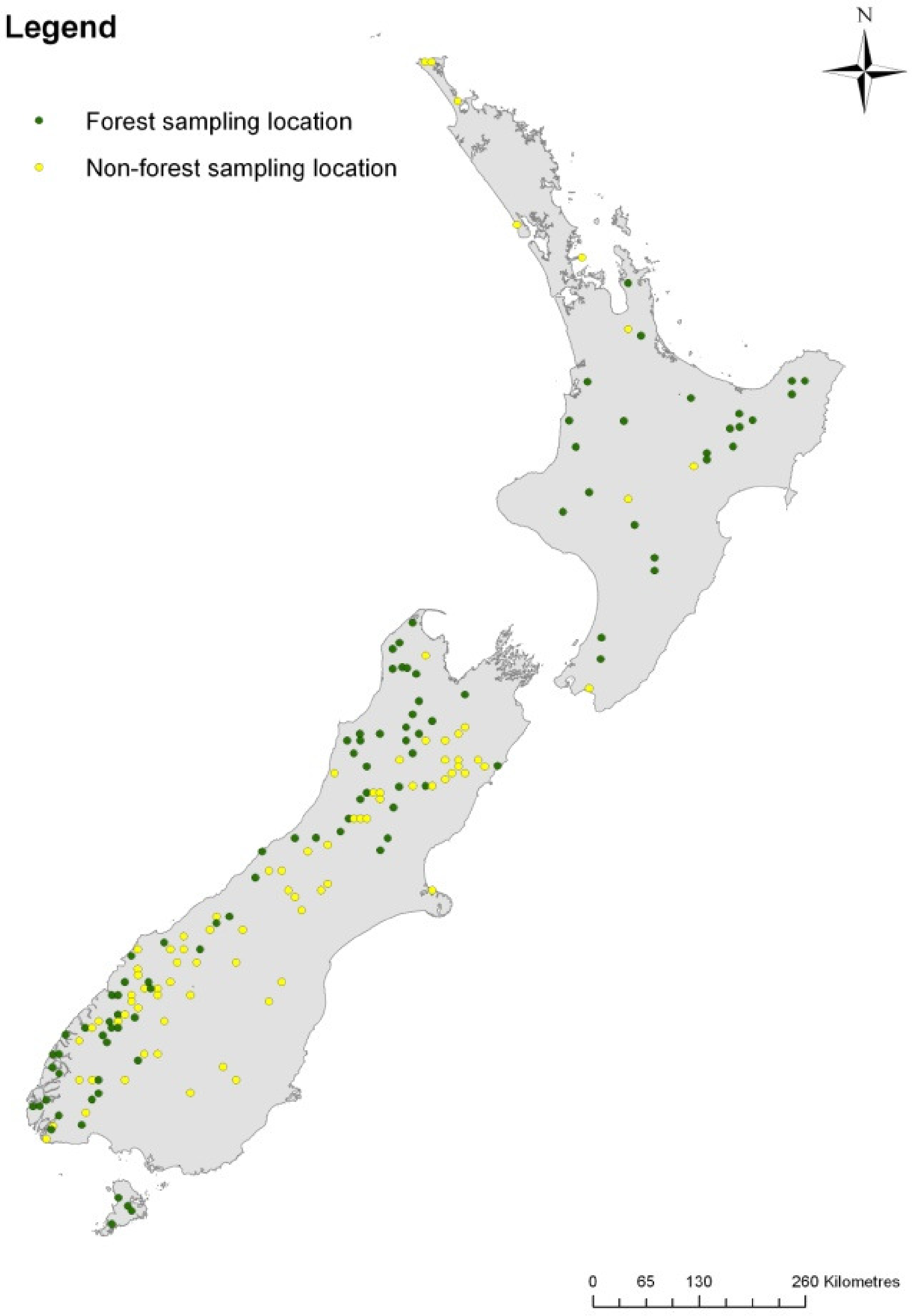

Supplement: S1 Fig — (PDF) [file pone.0127693.s001.pdf]
